# The VITAH Trial—Vitamin D Supplementation and Cardiac Autonomic Tone in Patients with End-Stage Kidney Disease on Hemodialysis: A Blinded, Randomized Controlled Trial

**DOI:** 10.3390/nu8100608

**Published:** 2016-09-28

**Authors:** Michelle C. Mann, Derek V. Exner, Brenda R. Hemmelgarn, David A. Hanley, Tanvir C. Turin, Jennifer M. MacRae, David C. Wheeler, Darlene Y. Sola, Sharanya Ramesh, Sofia B. Ahmed

**Affiliations:** 1 Department of Medicine, University of Calgary, Calgary, AB T2N 4Z6, Canada; mcmann@ucalgary.ca (M.C.M.); exner@ucalgary.ca (D.V.E.); brenda.hemmelgarn@albertahealthservices.ca (B.R.H.); dahanley@ucalgary.ca (D.A.H.); turin.chowdhury@albertahealthservices.ca (T.C.T.); jennifer.macrae@albertahealthservices.ca (J.M.M.); dsola@ucalgary.ca (D.Y.S.); sramesh@ucalgary.ca (S.R.); 2 Libin Cardiovascular Institute of Alberta, Calgary, AB T2N 4Z6, Canada; 3 Department of Community Health Sciences, University of Calgary, Calgary, AB T2N 4Z6, Canada; 4 Osteoporosis and Metabolic Bone Disease Centre, Calgary, AB T2T 5C7, Canada; 5 Department of Medicine, University College London, London NW3 2PF, UK; d.wheeler@ucl.ac.uk

**Keywords:** autonomic nervous system, chronic kidney disease, heart rate variability, hemodialysis, vitamin D

## Abstract

End-stage kidney disease (ESKD) patients are at increased cardiovascular risk. Vitamin D deficiency is associated with depressed heart rate variability (HRV), a risk factor depicting poor cardiac autonomic tone and risk of cardiovascular death. Vitamin D deficiency and depressed HRV are highly prevalent in the ESKD population. We aimed to determine the effects of oral vitamin D supplementation on HRV ((low frequency (LF) to high frequency (HF) spectral ratio (LF:HF)) in ESKD patients on hemodialysis. Fifty-six subjects with ESKD requiring hemodialysis were recruited from January 2013–March 2015 and randomized 1:1 to either conventional (0.25 mcg alfacalcidol plus placebo 3×/week) or intensive (0.25 mcg alfacalcidol 3×/week plus 50,000 international units (IU) ergocalciferol 1×/week) vitamin D for six weeks. The primary outcome was the change in LF:HF. There was no difference in LF:HF from baseline to six weeks for either vitamin D treatment (conventional: *p* = 0.9 vs. baseline; intensive: *p* = 0.07 vs. baseline). However, participants who remained vitamin D-deficient (25-hydroxyvitamin D < 20 ng/mL) after treatment demonstrated an increase in LF:HF (conventional: *n* = 13, ∆LF:HF: 0.20 ± 0.06, *p* < 0.001 vs. insufficient and sufficient vitamin D groups; intensive: *n* = 8: ∆LF:HF: 0.15 ± 0.06, *p* < 0.001 vs. sufficient vitamin D group). Overall, six weeks of conventional or intensive vitamin D only augmented LF:HF in ESKD subjects who remained vitamin D-deficient after treatment. Our findings potentially suggest that while activated vitamin D, with or without additional nutritional vitamin D, does not appear to improve cardiac autonomic tone in hemodialysis patients with insufficient or sufficient baseline vitamin D levels, supplementation in patients with severe vitamin D deficiency may improve cardiac autonomic tone in this higher risk sub-population of ESKD. Trial Registration: ClinicalTrials.gov, NCT01774812.

## 1. Introduction

Patients with end-stage kidney disease (ESKD) have 10–100 times greater risk of cardiovascular death compared to the general population, with sudden arrhythmic cardiac death (SCD) accounting for approximately 25% of all cardiovascular-related deaths [1]. Despite treatment of traditional cardiovascular risk factors, rates of cardiovascular mortality and SCD remain elevated in this patient population [2]. Suppressed heart rate variability (HRV), a measure of cardiac autonomic nervous system activity commonly depressed in ESKD patients [3], has been shown to predict SCD risk [4,5] and suggests that treatments aimed at normalizing HRV may translate into improved cardiac outcomes.

Vitamin D deficiency is common in ESKD [6,7] and is characterized by not only a deficiency in 25-hydroxyvitamin D, the barometer of vitamin D status, but also 1,25-dihydroxyvitamin D, the active form of vitamin D due to decreased renal 1-alpha hydroxylase activity [8]. Vitamin D deficiency has been shown to be associated with increased renin-angiotensin system (RAS) activity [9], abnormal HRV [10,11,12], and increased risk of cardiovascular mortality in patients with ESKD [6,7,13,14]. While, to date, no randomized trials have shown increased survival with vitamin D supplementation [15,16,17] in ESKD, treatment with the activated vitamin D analogue paricalcitol decreased cardiovascular-related hospitalizations compared to those treated with placebo [16,17]. We have previously shown that vitamin D3 supplementation is associated with normalization of HRV in healthy humans [18] and increased cardioprotective vagal activity in patients with Immunoglobluin A (IgA) nephropathy [19], suggesting that low vitamin D levels may mediate increased risk via the cardiac autonomic nervous system, though this has not been examined in a randomized-controlled trial.

The VITamin D supplementation and cardiac Autonomic tone in Hemodialysis (VITAH) trial was an investigator-initiated, blinded, randomized controlled trial, which tested the hypothesis that six-week treatment with intensive vitamin D [activated vitamin D (alfacalcidol) + nutritional vitamin D (ergocalciferol)] would normalize HRV measures of cardiac autonomic tone by enhancing cardiovagal activity in patients with ESKD requiring hemodialysis.

## 2. Experimental Section

### 2.1. Study Population

Details of the VITAH study design have been previously published [20]. In brief, participants were recruited in Calgary, Canada. Eligible patients were ≥18 years, and on outpatient hemodialysis three times per week for ≥3 months. Exclusion criteria included any major cardiovascular event (new onset arrhythmia, cardiovascular-related hospitalization or emergency room visit) within six months prior to enrollment, inability to cease non-study-related vitamin D, anticipated death, changes in dialysis modality or location or upcoming kidney transplantation within one year, active medical issues, or inability to provide informed consent. Demographic and clinical characteristics were collected prospectively.

### 2.2. Study Design 

The VITAH Trial was a 2 × 2 crossover, blinded, randomized-controlled trial. Subjects, investigators, health care providers, study coordinators and data analysts were blinded to treatment allocation. Subjects were recruited from January 2013 until October 2015 and underwent a four-week washout period of any type of vitamin D therapy. Subject identification codes and corresponding treatment allocations were developed by an independent biostatistician at the University of Calgary using random small block randomization methods, which were then held solely by the University of Calgary Research Pharmacy for the preparation and dispensing of unlabeled vitamin D blister packs. Identification codes were then allocated to each enrolled subject prospectively by the study coordinator, and provided to the Pharmacy for dispensing of the corresponding blister packs.

### 2.3. Randomization

**Figure A1:**
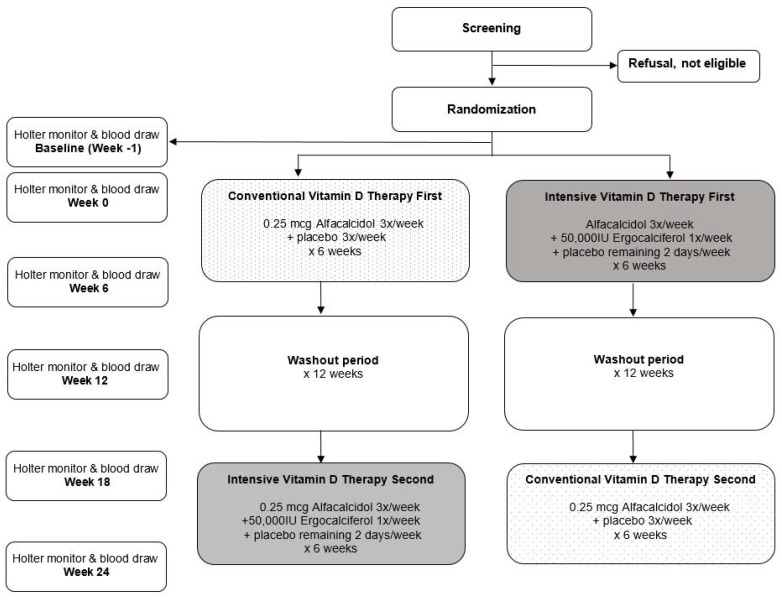
Overview of the VITAH Trial.

Subjects were subsequently randomized to one of two sequence arms; six weeks of conventional vitamin D therapy (0.25 mcg alfacalcidol plus placebo three times per week), followed by a 12 weeks washout, and six weeks of intensive vitamin D therapy (0.25 mcg alfacalcidol three times per week plus 50,000 IU of ergocalciferol once per week and placebo the remaining two days of the week), or vice versa for 24 weeks (Appendix Figure A1). All vitamin D treatments were taken orally at each hemodialysis session during the study period. The different vitamin D formulations and placebo tablets were encapsulated in identical 1 g soft gelatin capsules matching in appearance. Compliance with study treatment was monitored and recorded electronically by attending nursing staff at each hemodialysis unit.

### 2.4. Outcome Measures

Ambulatory electrocardiograph (ECG) monitors were applied to the subjects within the first hour of the first weekly dialysis run at baseline (oneweek prior to vitamin D intervention initiation) and every six weeks (Week 0, Week 6, Week 12, Week 18, Week 24) until trial termination (Appendix Figure A1). Ambulatory ECG data were collected for a minimum of 4 h and up to 24 h using a standard bipolar three-lead configuration (GE Healthcare, SEER MC; Milwaukee, WI, USA). Power spectral density analysis of heart rate variability (HRV), which transforms the electrocardiographic signals into measures of frequency domain (largely representing activity of the sympathetic and vagal activity) was carried out by computer-generated algorithms (MARS software program; GE Healthcare; Milwaukee, WI, USA). Autonomic activity was categorized into spectral bands: total power (TP), very-low frequency (VLF, 0.003–0.04 Hz), low-frequency (LF, 0.04–0.15 Hz) and high-frequency (HF, 0.15–0.4 Hz) domains (28, 35, 151–153). Absolute LF and HF parameters were not normally distributed; therefore, the values were squared and transformed into the natural logarithm (ln ms^2^), as well as converted to normalized units (nu) to account for potential background contribution from activity within the VLF frequency band. Time domain measurements were also generated using the beat-to-beat variation in normal R-R intervals, including the standard deviation of the normal waves (SDNN), standard deviation of the average normal wave (SDANN), and percentage of normal waves more than 50 ms difference between the immediate preceding normal wave (pNN50%) [21,22].

The primary outcome was the change in cardiac autonomic tone measured by the ratio of LF to HF frequency domain parameters (LF:HF) during the first 4 h of hemodialysis of the first hemodialysis session of the week, as the period of highest cardiovascular risk in hemodialysis patients occurs after the long interdialytic interval [23]. Though an increase in LF:HF ratio is associated with improved cardiac outcomes in non-ESKD populations [24], we hypothesized, based on our previous studies [18,19], that vitamin D supplementation would result in an overall decrease in LF:HF due a significant increase in HF. Furthermore, we also hypothesized that intensive vitamin D therapy would have a greater effect on LF:HF compared to conventional vitamin D alone. Time domain measures of HRV were also analyzed, but only if >12 h of ambulatory ECG data were available to ensure validity of these measures [21].

Pre-specified secondary end points included additional HRV measures, such as LF, HF, and time domain parameters. In addition, biochemical and dialysis adequacy measures were assessed at each six-week study visit throughout the trial.

### 2.5. Sample Size Determination

Sample size calculations were based on the expected change in the baseline LF:HF ratio. Post-myocardial infarction (MI) patients randomized to receive daily quinapril for 35 days demonstrated a 46% reduction in LF:HF from baseline [25]. Vitamin D has been shown to be a negative endocrine regulator of the RAS [9], and thus may act similarly to an angiotensin-converting enzyme inhibitor such as quinapril. Using a 2 × 2 crossover design and anticipating a 20% or larger reduction in LF:HF after intensive vitamin D treatment compared to conventional vitamin D treatment, we estimated 54 participants would be required (α = 0.05, β = 0.9). Assuming a 10% dropout rate, we aimed to recruit approximately 60 participants.

### 2.6. Patient Safety

Safety was evaluated as related to serious adverse events during the 24-week study period. An external data safety monitoring board independent of the steering committee operated under a formalized charter to monitor safety.

### 2.7. Study Approval

The study was approved by Health Canada and the University of Calgary Health Research Ethics Board (the ethical approval code: E-24846). All participants signed written informed consent prior to inclusion in the trial.

### 2.8. Statistical Analyses

All end-point data were collected and analyzed using the intention-to-treat principle. All data is presented as mean ± standard error (SE) unless otherwise indicated. The primary analysis tested associations between conventional and intensive vitamin D therapies and change in LF:HF and other HRV measures pre- vs. post- vitamin D within each treatment period utilizing a non-parametric paired *t*-test. Prompted by the results of the Correction of Vitamin D Deficiency in Critically Ill Patients (VITdAL-ICU) randomized clinical trial [26] demonstrating a lower hospital mortality with vitamin D supplementation only in the severely vitamin D deficiency subgroup of critically ill patients, a post hoc analysis was conducted with participants’ achieved 25-hydroxy vitamin D status after each six-week vitamin D treatment period (vitamin D deficient: <20 ng/mL; insufficient; 20–30 ng/mL; sufficient: >30 ng/mL) [27]. Changes in LF:HF and other measures of HRV pre- vs. post-vitamin D across these groups were compared with Kruskal–Wallis test and Tukey’s post hoc analysis. To further determine the relationships between HRV and vitamin D therapy, multivariate regression analysis was conducted. Age, sex treatment sequence, prior vitamin D supplementation, baseline calcium, parathyroid hormone, dialysis adequacy (kt/V), and β-blocker medication were tested as potential effect modifiers within the model, recognizing that the ability to detect significance was limited by sample size. Subgroup analyses were conducted for subjects with diabetes mellitus [28] and subjects on nocturnal hemodialysis [29]. Missing HRV values were imputed utilizing an expectation–maximization (EM) technique. Data from participants who completed at least the first treatment period before withdrawal (*n* = 4; three in intensive treatment, first arm, one in conventional treatment, first arm) were analyzed by paired *t*-test to assess the effect of that specific vitamin D therapy on HRV but were excluded from any further analyses. Statistical analyses were performed using SPSS (version 21; IBM, Armonk, NY, USA), with two-tailed significance levels of α = 0.05.

## 3. Results

### 3.1. Enrollment and Study Population

**Figure 1:**
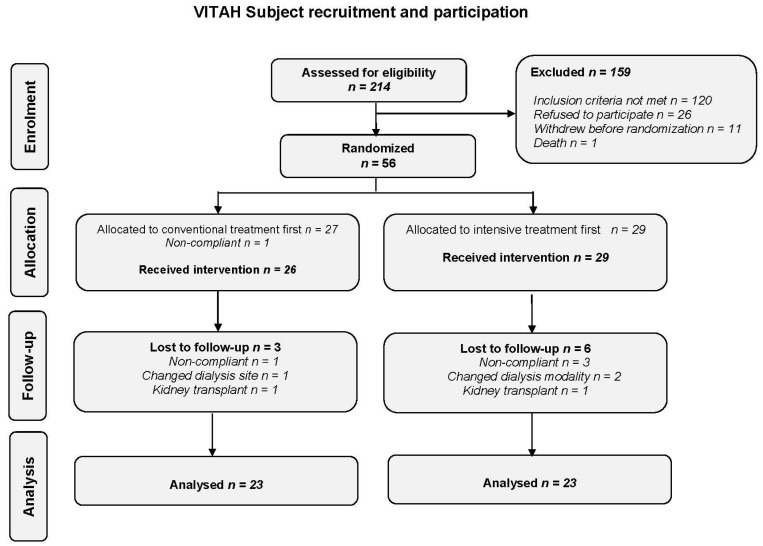
Consolidated Standard of Reporting Trials (CONSORT) diagram for trial recruitment and participation.

A total of 214 hemodialysis patients were screened of whom 56 participants were randomized from January 2013 to March 2015—27 to conventional treatment first and 29 to intensive treatment first (Figure 1). The trial was halted in March 2015 due to funding and time constraints; however, the achieved sample size allowed for the statistical power to be maintained above 80%. Demographics were balanced between treatment groups (Table 1). Participants were predominantly male, Caucasian, with the cause of ESKD due to diabetes or hypertension. Most participants were receiving medications interrupting the RAS, and the use of beta-blockers was balanced between groups. The majority of participants underwent daytime dialysis, and more than half were on some form of vitamin D supplementation prior to enrollment in the study. No participants were vitamin D sufficient at baseline, with approximately half of the participants vitamin D insufficient (*n* = 31) and half vitamin D deficient (*n* = 25). All participants demonstrated low levels of serum 1,25-dihydroxy vitamin D [18,19]. Measures of circulating components of the RAS were markedly elevated [18] but similar between groups. Post-dialysis systolic blood pressure was lower in the intensive vitamin D group (*p* = 0.03), but blood pressure was well controlled in both groups. All measures of HRV were depressed but were similar between groups and comparable to published measures in the ESKD population [3,24,28,30].

### 3.2. Cardiac Autonomic Tone and Mineral Metabolism Responses

**Figure 2:**
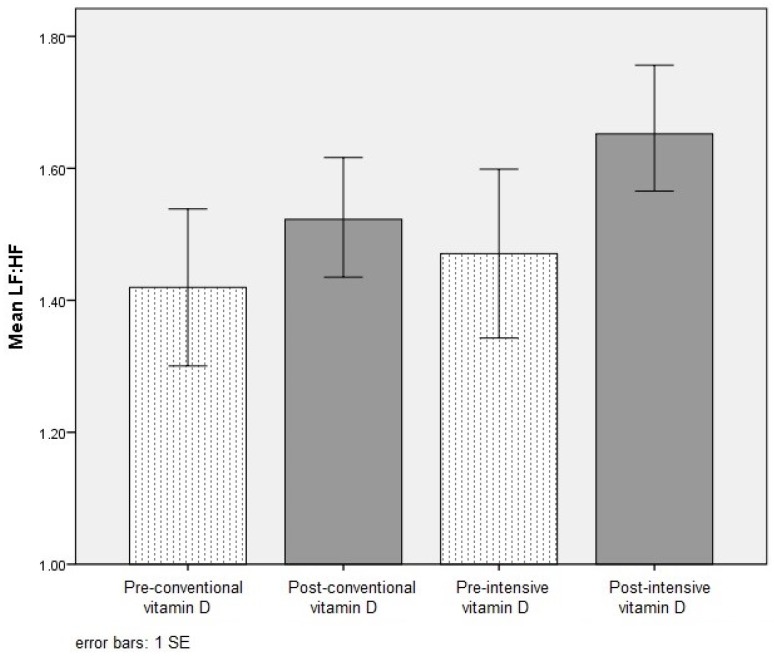
Low frequency to high frequency ratio (LF:HF) responses to vitamin D supplementation.

A similar number of participants in each group reached the final visit at 24 weeks (conventional-first, 85.2% vs. intensive-first, 79.3%, *p* = 0.9). The primary end point was change in overall cardiac autonomic tone as measured by LF:HF at six weeks, which did not significantly differ from baseline after either vitamin D treatment period (Table 2). LF:HF increased but not significantly at week 6 after both conventional (*p* = 0.9) and intensive (*p* = 0.07) vitamin D treatments compared to baseline (Figure 2). Neither LF nor HF changed significantly in both the conventional (LF nu, *p* = 0.8; HF nu, *p* = 0.9) and intensive (LF nu, *p* = 0.8; HF nu, *p* = 0.7) vitamin D groups. Other HRV measures (SDNN, SDANN, pNN50%) were highly variable and did not differ between the two vitamin D therapies (Table 2).

**Figure A2:**
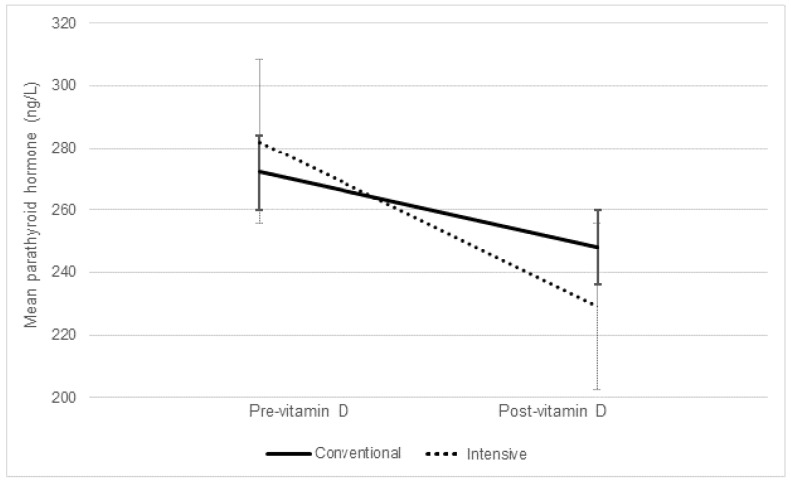
Parathyroid hormone response to vitamin D supplementation.

A number of participants remained vitamin D deficient (<20 ng/mL 25-hydroxyvitamin D) following either type of vitamin D therapy (Appendix Table A1). The majority of subjects achieved a vitamin D level within the vitamin D insufficient range with vitamin D therapy with only a minority of subjects achieving vitamin D sufficiency. Nursing records depicting dispersal of study medication at each dialysis session as well as the non-statistically significant dose-dependent reduction in parathyroid hormone (PTH) during both vitamin D treatment periods provided evidence of compliance with both vitamin D therapies (Appendix Figure A2).

In response to conventional treatment, neither 25-hydroxy (*p* = 0.9) nor 1,25-dihydroxy (*p* = 0.8) vitamin D levels changed from baseline. Conversely, following intensive treatment, 25-hydroxyvitamin D levels rose significantly (*p* = 0.001), although 1,25-dihydroxy vitamin D levels did not differ (*p* = 0.7) from baseline. Mineral metabolism parameters did not change from baseline with either conventional or intensive vitamin D treatment, and PTH remained within range (Appendix Figure A2) [27]. Both conventional and intensive vitamin D supplementation elicited similar, non-significant effects on measures of circulating RAS components or blood pressure control (Table 2).

### 3.3. Post Hoc Analyses

**Figure 3:**
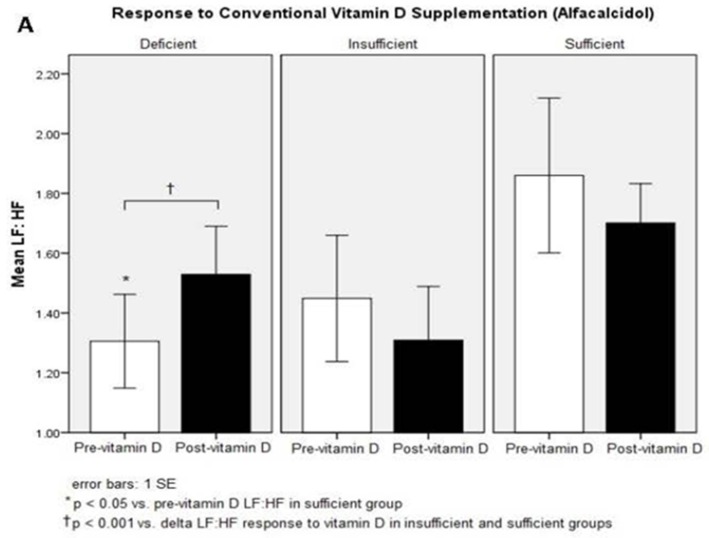

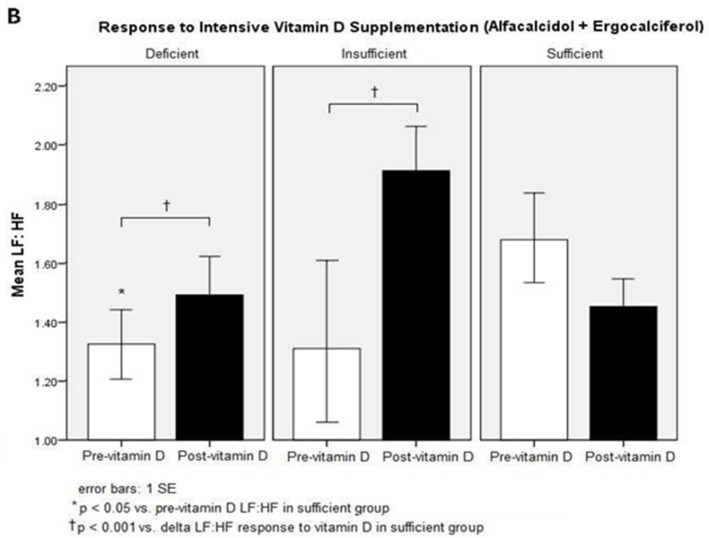
LF:HF responses to vitamin D supplementation, stratified by achieved vitamin D status; (**A**) responses to alfacalcidol alone; (**B**) responses to alfacalcidol and ergocalciferol combined supplementation.

In post hoc analyses, participants who remained vitamin D-deficient after either vitamin D treatment demonstrated significantly lower baseline LF:HF compared to subjects who achieved sufficient vitamin D status (*p* = 0.01 after conventional treatment; *p* = 0.03 after intensive treatment). Stratification by post-treatment vitamin D status demonstrated that HRV responses to vitamin D supplementation differed significantly by group. In response to conventional vitamin D therapy, participants who remained vitamin D deficient showed a trend towards an increased LF:HF (*p* = 0.06), an increase not observed in subjects who achieved vitamin D levels within the insufficient and sufficient ranges (*p* < 0.001 vs. deficient vitamin D group) (Appendix Table A1, Figure 3). A similar trend was observed after six weeks of intensive vitamin D treatment (Figure 3). Those participants who achieved vitamin D sufficiency in response to intensive vitamin D therapy demonstrated an increase in HF (*p* = 0.05 pre- vs. post supplementation) (Appendix Table A1). Vitamin D therapy-dependent changes in measures of mineral metabolism and RAS activity were not significantly different between any of the three vitamin D status groups. Treatment sequence, prior vitamin D supplementation, 25-hydroxy vitamin D level, and all other potential modifiers had no significant effect on outcomes.

### 3.4. Subgroup Analyses

Diabetic subjects (*n* = 17) and nocturnal dialysis subjects (*n* = 6) displayed similar measures of mineral metabolism, RAS, blood pressure (BP), and HRV pre- and post-conventional and intensive vitamin D treatment, with similar trends in changes of HRV measures post-vitamin D as observed in the primary analyses.

### 3.5. Adverse Events

There was no difference in the overall incidence of adverse events between groups (conventional-first, 7.4% vs. intensive-first, 6.9%, *p* = 0.9), and no adverse events were judged to be related to vitamin D therapy. There was one episode of gastrointestinal upset and one hospitalization due to upper airway compromise in the conventional-first group, one incident of gastrointestinal upset and one episode of increased lower leg edema in the intensive-first group. No participants withdrew from the study because of adverse events. There were no deaths during the study period or within 30 days of study completion.

## 4. Discussion

Vitamin D deficiency is associated with cardiovascular risk in ESKD [6,7,13,14], and limited data suggests that supplementation may mitigate risk in this population [16,17] via favourable alterations in HRV [18,19]. In this blinded, randomized controlled trial, administration of both nutritional and activated (intensive) vitamin D supplementation compared with activated vitamin D alone (conventional) did not alter HRV among patients with ESKD on thrice weekly hemodialysis. However, in post hoc analysis, a significant increase in LF:HF ratio was observed in response to both vitamin D supplementation treatments exclusively in subjects with the poorest vitamin D status, suggesting a potential cardiovascular benefit of vitamin D therapy in ESKD patients with the lowest 25-hydroxy vitamin D levels.

Impaired HRV is a valid surrogate marker of cardiovascular risk, and is thought to play a central role in the risk of SCD in patients with ESKD [5,24,30]. However, reduced HRV measures remain relatively under-recognized as a predictor of adverse cardiovascular events in clinical practice [24]. Depressed HRV is extremely common in the ESKD population [3,4,5,24], and commonly presents as withdrawal of vagal activity in conjunction with an increase in the sympathetic input [3]. Consequently, interventions favourably altering cardiac autonomic tone could potentially hold considerable therapeutic benefit.

Observational studies suggest that low levels of vitamin D are associated with increased risk of SCD in ESKD patients [6,7,13,14], and vitamin D deficiency is extremely common in the ESKD population [7,8,14]. We have previously shown that in both healthy volunteers and subjects with IgA nephropathy, oral vitamin D3 supplementation was associated with normalization of autonomic tone due to significantly increased vagal (HF) activity in response to angiotensin II [18,19], a chronically upregulated hormone in the ESKD population. As a result of this increase in vagal tone, there was an overall decrease in the LF:HF ratio. However, contrary to our hypothesis, we observed no difference in LF:HF with conventional (i.e., activated vitamin D alone) treatment and a trend towards an increase in cardiosympathovagal balance (LF:HF) with intensive (activated and nutritional) vitamin D supplementation. Furthermore, the increase in LF:HF attributed to vitamin D supplementation appeared to be driven primarily by participants with the lowest vitamin D status. Interestingly, and in support of our a priori hypothesis, an increase in HF with vitamin D supplementation was observed but only in the participants who achieved vitamin D sufficient status. The similar cardiovagal response to vitamin D supplementation in this sufficient group compared to healthy subjects [18] and subjects with early CKD [19] suggests that these subjects may have been in better general health to elicit a comparable response. We also speculate that the differences observed with vitamin supplementation on cardiac autonomic tone between the previous studies and the VITAH study are due to differences in vitamin D formulations and study populations. This phenomenon, whereby outcomes differ in the ESKD population compared to other populations, is not uncommon [31,32].

The observation that vitamin D therapy had an effect on overall LF:HF only in subjects with low vitamin D status and not those with vitamin D sufficiency is suggestive of a threshold effect of the benefit of vitamin D, potentially similar to the subgroup analysis results of the VITdAL-ICU clinical trial [26], in which only the severely vitamin D deficient subgroup of critically ill subjects who were provided with vitamin D supplementation showed a lower mortality risk. Interestingly, there were no significant differences in HRV to vitamin D treatment in the stratified groups when comparing conventional and intensive vitamin D supplementation, implying that the observed effects on cardiac autonomic nervous system activity are principally driven by the activated 1,25-dihydroxy vitamin D metabolite (via 25-hydroxylation of alfacalcidol). Our data thus provide a potential physiological explanation for the findings reported by Wolf and colleagues [6]. While vitamin D status was associated with all-cause and cardiovascular mortality in ESKD patients on hemodialysis in this large cross-sectional study, the association was abolished in those patients on activated vitamin D supplementation, suggesting that 1,25-dihydroxyvitamin D status, rather than 25 hydroxyvitamin D status, determines risk.

## 5. Limitations

To date, there are no adequate clinical trials demonstrating a causal relationship between vitamin D and adverse cardiovascular outcomes in CKD and ESKD, with the majority of existing trials primarily assessing the impact of vitamin D on biochemical outcomes rather than patient-level outcomes [33]. However, compelling evidence from observational studies suggests that vitamin D plays a role in cardiovascular risk in the ESKD population [7,13,14]. While our study is the first to assess a causal relationship between vitamin D supplementation and a surrogate marker of cardiovascular risk, it was not without limitations. First, the intervention periods for both vitamin D therapies were only six weeks in length, and thus any effects observed may not be representative of long-term exposure to vitamin D treatment, although our results add to the literature demonstrating the short-term safety of intensive vitamin D treatment with both nutritional and activated vitamin D. Total 25-hydroxy vitamin D levels are currently accepted as the barometer of vitamin D status [34]; however, it has been suggested that the free or bioavailable fraction of vitamin D provides a more reliable assessment of the vitamin D being used for cellular processes. Furthermore, the notion that vitamin D may have local roles within the nervous system is relatively new; nevertheless, recent review papers have shown strong evidence to support our hypotheses and results herein [35,36,37,38]. A total of nine subjects dropped out throughout the duration of the study, mostly due to non-compliance with the vitamin D treatment or changes in hemodialysis site and modality. Fortunately, the study characteristics of remaining subjects in both treatment arms (*n* = 23 in each) remained similar, and we found no evidence of the impact of subject attrition within our patient sample or results (Table 1). Our primary outcome was a surrogate marker of risk, but changes in HRV are independently associated with corresponding changes in cardiovascular risk [39]. This study was conducted in a single centre and recruited patients on a voluntary basis. It is possible that healthy volunteer bias may have altered the results of our study in which those subjects who enrolled and completed the trial are healthier than the average hemodialysis patient, which may limit the generalizability of our findings. However, despite its modest size, the VITAH trial represents the largest prospective blinded, randomized, placebo-controlled trial of intensive vitamin D therapy in ESKD patients to date.

## 6. Conclusions

In conclusion, we found that six weeks of treatment with either conventional or intensive vitamin D did not alter HRV measures in patients with end-stage kidney disease on hemodialysis. Increased LF:HF, which may translate into decreased cardiovascular risk [24], was observed in the vitamin D deficient subgroup; however, this finding should be considered hypothesis generating and requires further study.

## Figures and Tables

**Table 1:** Baseline characteristics of study subjects.

	All Subjects (*n* = 56)	Conventional Vitamin D Therapy First (*n* = 27)	Intensive Vitamin D Therapy First (*n* = 29)
Age	66 ± 2	67 ± 2	66 ± 3
Male sex (%)	41 (73%)	19 (70%)	22 (76%)
Race			
Caucasian	33 (59%)	15 (56%)	18 (62%)
Asian	17 (30%)	9 (33%)	8 (28%)
Black	1 (2%)	0 (0%)	1 (3%)
Other	5 (9%)	3 (11%)	2 (7%)
Cause of ESKD			
Diabetes	17 (31%)	9 (33%)	8 (28%)
Hypertension	11 (20%)	4 (15%)	7 (24%)
Glomerulonephritis	5 (9%)	1 (4%)	4 (14%)
Unknown	23 (41%)	13 (48%)	10 (34%)
Dialysis vintage (months)	38 ± 3	42 ± 4	34 ± 4
History of CVD or related events (%)	23 (41%)	10 (37%)	13 (45%)
Diabetes (%)	17 (30%)	9 (33%)	8 (28%)
Dialysis schedule (%)			
Morning	21 (38%)	9 (33%)	12 (41%)
Afternoon	23 (41%)	8 (30%)	15 (52%)
Evening	6 (11%)	6 (22%)	0 (0%)
Nocturnal	6 (11%)	4 (15%)	2 (7%)
Medications			
ACE-inhibitors/ARBs	41 (73%)	19 (70%)	22 (76%)
Statins	18 (32%)	9 (33%)	9 (31%)
β-blockers	5 (9%)	2 (7%)	3 (10%)
Cinacalcet	5 (9%)	3 (11%)	2 (7%)
Current vitamin D therapy			
Alfacalcidol	9 (16%)	7 (25%) 4 (15%)	2 (7%)
Calcitriol	10 (18%)	5 (19%)	6 (21%)
Cholecalciferol	9 (16%)	1 (4%)	4 (14%)
Combination (Calcitriol + Cholecalciferol)	4 (7%)		3 (10%)
25(OH) Vitamin D (ng/mL) ^†^	21 ± 4	20 ± 5	22 ± 5
1,25(OH)_2_ Vitamin D (pg/mL) ^†^	49 ± 5	56 ± 9	43 ± 5
Serum Calcium (mmol/L)	2.15 ± 0.05	2.11 ± 0.06	2.19 ± 0.08
Serum Phosphate (mmol/L)	1.57 ± 0.07	1.44 ± 0.06	1.62 ± 0.09
Serum PTH (ng/L)	241 ± 29	216 ± 49	263 ± 34
Renin (mg/mL/s)	2.8 ± 0.36	1.91 ± 0.50	3.21 ± 1.5
Ang II (pg/mL)	26.9 ± 2.9	22.6 ± 3.7	30.9 ± 4.4
Aldosterone (pmol/L)	766 ± 223	3330 ± 888	2241 ± 950
Post-dialysis heart rate (bpm)	69 ± 2	69 ± 2	68 ± 3
Post-dialysis SBP (mmHg)	122 ± 6	129 ± 8	110 ± 8 *
Post-dialysis DBP (mmHg)	63 ± 2	66 ± 3	60 ± 4
Ultrafiltration volume (mL)	1726 ± 133	1543 ± 168	2016 ± 202
Kt/V	1.27 ± 0.08	1.33 ± 0.11	1.17 ± 0.11
HRV Measures			
LF:HF	1.40 ± 0.08	1.34 ± 0.12	1.41 ± 0.11
LF (ms^2^)	586 ± 108	557 ± 215	597 ± 215
LF (*ln* ms^2^)	5.05 ± 0.24	4.98 ± 0.42	5.10 ± 0.25
LF (nu)	52 ± 3	50 ± 5	53 ± 4
HF (ms^2^)	312 ± 62	313 ± 116	272 ± 51
HF (*ln* ms^2^)	4.56 ± 0.22	4.64 ± 0.37	4.14 ± 0.25
HF (nu)	34 ± 2	35 ± 3	33 ± 3
SDNN (ms) ^‡^	88 ± 13	89 ± 11	77 ± 8
SDANN (ms) ^‡^	72 ± 14	72 ± 11	69 ± 10
pNN50% ^‡^	9.2 ± 2.5	10.3 ± 4.1	6.15 ± 2.1

ESKD: end stage kidney disease; CVD: cardiovascular disease; ACE: angiotensin-converting enzyme; ARB: angiotensin receptor blocker; PTH: parathyroid hormone; AngII: angiotensin II; Epi: epinephrine; NE: norepinephrine; SBP: systolic blood pressure; DBP: diastolic blood pressure; VLF: very-low frequency; LF: low-frequency; HF: high-frequency; SDNN: standard deviation of the normal NN interval; SDANN: standard average deviation of the normal NN interval; pNN50%: percentage of NN intervals greater than 50 ms different than the preceding NN wave. Data is presented as mean ± SE * *p* < 0.05 vs. conventional vitamin D first group; ^†^ Post four-week washout period including *n* = 31 participants previously on vitamin D supplementation; ^‡^ Time domain measures used only in subjects with ≥12 h Holter recording, *n* = 14 in conventional vitamin D therapy first group, *n* = 17 in intensive vitamin D therapy first group.

**Table 2:** Responses to conventional and intensive vitamin D supplementation.

	Conventional Vitamin D *n* = 46		Intensive Vitamin D *n* = 46	
	Pre	Post	Pre	Post
25(OH) Vitamin D (ng/mL)	22 ± 4	23 ± 5	21 ± 4	33 ± 5 *^,†^
1,25(OH)_2_ Vitamin D (pg/mL)	50 ± 7	42 ± 4	36 ± 7	49 ± 5
Serum Calcium (mmol/L)	2.2 ± 0.03	2.2 ± 0.04	2.3 ± 0.03	2.3 ± 0.04
LF:HF	1.42 ± 0.09	1.50 ± 0.08	1.44 ± 0.12	1.63 ± 0.08
LF (ms^2^)	498 ± 122	565 ± 162	585 ± 151	589 ± 162
LF (*ln* ms^2^)	5.22 ± 0.31	5.57 ± 0.27	5.32 ± 0.24	5.45 ± 0.24
LF (nu)	56 ± 3	59 ± 3	57 ± 4	62 ± 3
HF (ms^2^)	353 ± 89	287 ± 78	359 ± 84	288 ± 73
HF (*ln* ms^2^)	5.07 ± 0.28	4.69 ± 0.24	5.11 ± 0.75	5.00 ± 0.24
HF (nu)	33 ± 2	31 ± 2	32 ± 2	29 ± 3
SDNN (ms) ^‡^	84 ± 8	68 ± 5	76 ± 6	73 ± 6
SDANN (ms) ^‡^	64 ± 8	47 ± 5	56 ± 6	54 ± 6
pNN50% ^‡^	10.4 ± 2.6	7.8 ± 2.0	7.5 ± 1.9	9.4 ± 2.6
Serum Phosphate (mmol/L)	1.4 ± 0.08	1.6 ± 0.20	1.6 ± 0.06	1.5 ± 0.06
Serum PTH (ng/L)	260 ± 32	240 ± 20	295 ± 26	229 ± 18
Renin (mg/mL/h)	3.3 ± 1.1	3.1 ± 1.1	3.9 ± 0.9	3.0 ± 0.8
Ang II (pg/mL)	28 ± 4	21 ± 2	26 ± 2	23 ± 3
Aldosterone (pmol/L)	756 ± 207	448 ± 164	828 ± 165	779 ± 177
Post-dialysis heart rate (bpm)	69 ± 2	66 ±1	68 ± 2	67 ± 2
Post-dialysis SBP (mmHg)	130 ± 4	130 ± 3	119 ± 4	128 ± 3
Post-dialysis DBP (mmHg)	64 ± 2	61 ± 2	66 ± 4	61 ± 2
Kt/V	1.36 ± 0.05	1.37 ± 0.05	1.32 ± 0.05	1.40 ± 0.07

PTH: parathyroid hormone; AngII: angiotensin II; SBP: systolic blood pressure; DBP: diastolic blood pressure; VLF: very-low frequency; LF: low-frequency; HF: high-frequency; SDNN: standard deviation of the normal NN interval; SDANN: standard average deviation of the normal NN interval; pNN50%: percentage of NN intervals greater than 50 ms different than the preceding NN wave; Data is presented as mean ± SE * *p* < 0.05 vs. pre-vitamin D response; ^†^ *p* < 0.05 vs. conventional vitamin D treatment; ^‡^ Time domain measures used only in subjects with ≥12 h Holter recording, *n* = 14 in conventional vitamin D therapy first group, *n* = 17 in intensive vitamin D therapy first group.

## Appendix

**Table A1:** Response to conventional and intensive vitamin D supplementation, stratified by achieved vitamin D status.

	Conventional Vitamin D *n* = 46						Intensive Vitamin D *n* = 46					
	Deficient <50 nmol/L 25(OH)D *n* = 13		Insufficient 50–75 nmol/L 25(OH)D *n* = 27		Sufficient >75 nmol/L 25(OH)D *n* = 7		Deficient <50 nmol/L 25(OH)D *n* = 8		Insufficient 50–75 nmol/L 25(OH)D *n* = 23		Sufficient >75 nol/L 25(OH)D *n* = 15	
	Pre	Post	Pre	Post	Pre	Post	Pre	Post	Pre	Post	Pre	Post
25(OH)	41 ± 4 †	45 ± 3 *	56 ± 5	65 ± 5	61 ± 9	91 ± 4 *,†,ǂ	30 ± 4 †	49 ± 4 *	49 ± 5	59 ± 2	53 ± 5	104 ± 6 *,†,ǂ
1,25 (OH)_2_	52 ± 18	52 ± 6	51 ± 8	50 ± 6	49 ± 5	59 ± 9	26 ± 6 †	21 ± 8 *	37 ± 5	50 ± 12	38 ± 4	50 ± 6
LF:HF	1.31 ± 0.16 †	1.53 ± 0.16 ǂ	1.44 ± 0.21	1.36 ± 0.10	1.86 ± 0.26	1.65 ± 0.18	1.32 ± 0.15†	1.49 ± 0.12 ǂ	1.29 ± 0.38	1.87 ± 0.22 *,ǂ	1.66 ± 0.14	1.43 ± 0.10
LF (nu)	52 ± 6 †	60 ± 6	59 ± 6 †	56 ± 8	76 ± 6	66 ± 5	52 ± 7	57 ± 7	47 ± 8	69 ± 5	66 ± 4	62 ± 3
HF (nu)	35 ± 4	36 ± 4	37 ± 5	42 ± 3	25 ± 5	29 ± 6	37 ± 5	32 ± 4	39 ± 8	25 ± 4	29 ± 3	36 ± 2 *

25(OH): 25-hydroxy vitamin D (ng/mL): 1,25(OH)2 ;1,25-dihydroxy vitamin D (pg/mL); LF: low-frequency; HF: high-frequency. Data is presented as mean ± SE, * *p* < 0.05 vs. pre-vitamin D response, † *p* < 0.05 vs. sufficient vitamin D group at same time point, ǂ *p* < 0.05 vs. delta (post–pre vitamin D response) vs. other vitamin D group.
